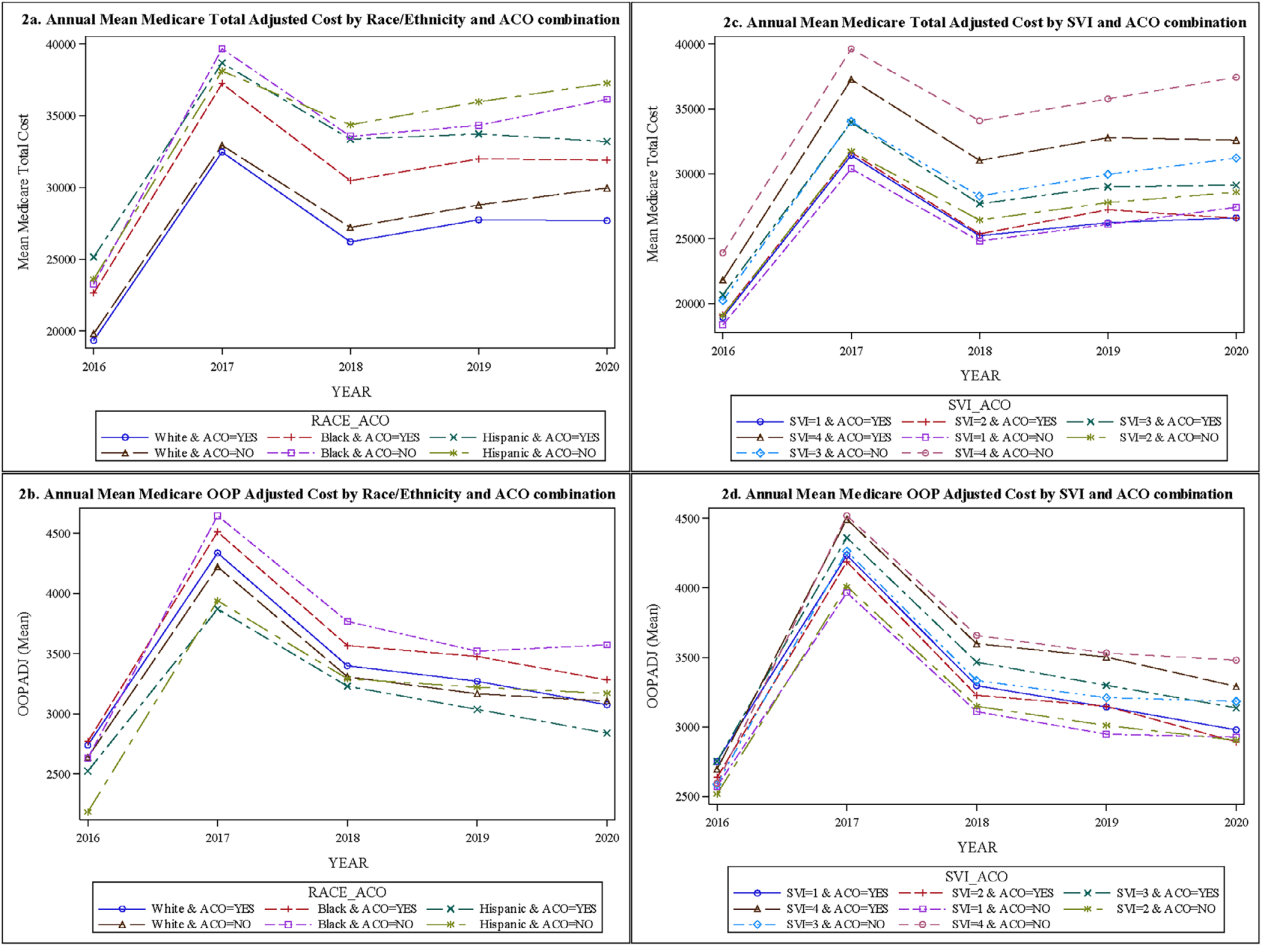# Accountable Care Organizations and Medicare Payments for Dementia Patients Across Race and Social Vulnerability

**DOI:** 10.1002/alz.084602

**Published:** 2025-01-09

**Authors:** Jie Chen

**Affiliations:** ^1^ University of Maryland School of Public Health, College Park, MD USA

## Abstract

**Background:**

Black and Hispanic ADRD patients often incur substantially higher costs related to Alzheimer’s disease and related dementia (ADRD). Neighborhood‐level vulnerabilities are also linked to increased risks of adverse health conditions. Emerging evidence has suggested that Accountable Care Organizations (ACOs) work effectively to promote ADRD care. This study aims to investigate the variations in Medicare cost for ADRD by race and ethnicity and social vulnerabilities and to further examine the variation by beneficiaries’ enrollment in ACOs among ADRD patients.

**Method:**

Using the longitudinal datasets of the 2016 ‐ 2020 Medicare Beneficiary Summary File, our study examined Medicare payments for patients newly diagnosed with ADRD and tracked these payments for the year preceding the diagnosis and for the subsequent three years. We calculated annual Medicare and beneficiary payments (OOP) for Medicare Fee‐For‐Service beneficiaries aged 65 or older. In addition, using beneficiaries’ zip codes, we merged the Medicare claims data with the CDC’s Social Vulnerability Index (SVI). Finally, we linked the dataset with the Medicare Shared Savings Program ACO to measure beneficiary‐level ACO enrollment.

**Results:**

Black patients newly diagnosed with ADRD and patients living in the most vulnerable areas encountered the highest total cost and OOP. Black patients enrolled in ACOs had a total cost of $37,244.31 in their diagnosis year, compared to $39,678.12 for those not enrolled in ACOs. This cost discrepancy sustained and increased to $4,225 three years post‐diagnosis. Differences in total costs among Hispanic and White ADRD patients by ACO affiliation were nominal at the diagnosis year. However, after three years post‐diagnosis, these differences became more pronounced. ADRD patients living in the most vulnerable areas without ACO affiliations faced the highest total cost ($39,635.83) than their ACO counterparts ($37,283.09) and those living in other SVIs.

**Conclusions:**

Results indicated that Black and Hispanic ADRD patients and ADRD patients living in areas with higher social vulnerability would gain more from ACO enrollment compared to their counterparts. Results of the study shed light on whether CMS innovation models like ACO and GUIDE can and where they can target to bridge gaps in ADRD care and promote more health equity.